# Identification of new potential molecular actors related to fiber quality in flax through Omics

**DOI:** 10.3389/fpls.2023.1204016

**Published:** 2023-07-17

**Authors:** Malika Chabi, Estelle Goulas, Dmitry Galinousky, Anne-Sophie Blervacq, Anca Lucau-Danila, Godfrey Neutelings, Sébastien Grec, Arnaud Day, Brigitte Chabbert, Katharina Haag, Jörg Müssig, Sandrine Arribat, Sébastien Planchon, Jenny Renaut, Simon Hawkins

**Affiliations:** ^1^ Université de Lille, CNRS, UMR 8576 - UGSF - Unité de Glycobiologie Structurale et Fonctionnelle, Lille, France; ^2^ Université de Lille, UMRT 1158 BioEcoAgro, Institut Charles Viollette, Lille, France; ^3^ Fibres Recherche Développement, Technopole de l’Aube en Champagne – Hôtel de Bureaux 2, 2 rue Gustave Eiffel, CS 90601, Troyes, France; ^4^ Université de Reims Champagne-Ardenne, INRAE, FARE, UMR A 614, Reims, France; ^5^ Fraunhofer-Institute for Manufacturing Technology and Advanced Materials IFAM, Bremen, Germany; ^6^ The Biological Materials Group, HSB – City University of Applied Sciences, Bremen, Germany; ^7^ Department of Environmental Research and Innovation, Luxembourg Institute of Science and Technology, Esch-sur-Alzette, Luxembourg

**Keywords:** flax (*Linum usitatissimum* L.), proteomics, transcriptomics, cell wall, fiber, retting, quality

## Abstract

One of the biggest challenges for a more widespread utilization of plant fibers is to better understand the different molecular factors underlying the variability in fineness and mechanical properties of both elementary and scutched fibers. Accordingly, we analyzed genome-wide transcription profiling from bast fiber bearing tissues of seven different flax varieties (4 spring, 2 winter fiber varieties and 1 winter linseed) and identified 1041 differentially expressed genes between varieties, of which 97 were related to cell wall metabolism. KEGG analysis highlighted a number of different enriched pathways. Subsequent statistical analysis using Partial Least-Squares Discriminant Analysis showed that 73% of the total variance was explained by the first 3 X-variates corresponding to 56 differentially expressed genes. Calculation of Pearson correlations identified 5 genes showing a strong correlation between expression and morphometric data. Two-dimensional gel proteomic analysis on the two varieties showing the most discriminant and significant differences in morphometrics revealed 1490 protein spots of which 108 showed significant differential abundance. Mass spectrometry analysis successfully identified 46 proteins representing 32 non-redundant proteins. Statistical clusterization based on the expression level of genes corresponding to the 32 proteins showed clear discrimination into three separate clusters, reflecting the variety type (spring-/winter-fiber/oil). Four of the 32 proteins were also highly correlated with morphometric features. Examination of predicted functions for the 9 (5 + 4) identified genes highlighted lipid metabolism and senescence process. Calculation of Pearson correlation coefficients between expression data and retted fiber mechanical measurements (strength and maximum force) identified 3 significantly correlated genes. The genes were predicted to be connected to cell wall dynamics, either directly (Expansin-like protein), or indirectly (NAD(P)-binding Rossmann-fold superfamily protein). Taken together, our results have allowed the identification of molecular actors potentially associated with the determination of both *in-planta* fiber morphometrics, as well as *ex-planta* fiber mechanical properties, both of which are key parameters for elementary fiber and scutched fiber quality in flax.

## 1 Introduction

Climate change is becoming a major concern, and strong decisions will have to be taken in the coming years to reduce global CO_2_ production. In this context, a major challenge will be finding environmentally-friendly replacements for the fossil-fuel-based composites used in everyday life. Renewable materials containing plant fibers (natural fiber composites: NFCs) offer the double advantage of having a low environmental impact during production and being compatible with material end-of-life issues. Beyond the environmental arguments, natural fibers from plants have comparable and/or superior properties to synthetic fibers leading to increased use in several different industries, including building, transportation and sports (Bourmaud et al., 2018).

Flax (*Linum usitatissinum* L.) is an economically important fiber species grown for its long cellulose-rich fibers that are grouped together in fiber bundles located in the outer stem tissues, as well as for its seeds (Esau, 1943). It is one of the oldest cultivated plants and the current *L. usitatissinum* species is probably derived from pale flax *L. bienne* (Diederichsen and Hammer, 1995; Rahman and Hoque, 2023). There is evidence of flax fiber use in Neolithic cultures, and plants were later grown in Egypt about 6,000 years ago (Zohary and Hopf, 2004). However, whether flax selection was driven mainly for seed oil content or for fiber yield in the stems is still a matter of debate.

Previous work on flax-based NFCs has shown that their properties depend on several factors, including processing technique, polymer type and fiber quality (Haag et al., 2017). Defining “fiber quality” is difficult since it is related to what is really meant by the term “fiber” (Wrobél-Kwiatowska et al., 2010). The word fiber can refer to individual fiber cells - the botanical “elementary fibers” - or to groups of several elementary fibers glued together in which case the term “scutched fiber” is sometimes used. The fineness and the mechanical properties of both elementary and scutched fibers are key parameters used to define fiber quality, especially for textiles and/or NFCs. These parameters are linked to the plant genotype, environmental conditions, and the degree of elementary fiber separation obtained during the retting, scutching, and cleaning processes (Chabbert et al., 2020).

At the cellular level, both fineness and mechanical properties are related to the structural composition and the architecture of the fiber composite cell wall consisting of the middle lamella, the primary and gelatinous secondary cell wall characterized by high amounts of crystalline cellulose and very low lignin levels (Morvan et al., 2003; Day et al., 2005; Bourmaud et al., 2013). Although some broad correlations can currently be made between cell wall structure and fiber quality (e.g., negative relationship between lignin content and textile fiber quality), it is likely that many other more or less subtle variations in the cell wall structure and metabolism could contribute to differences in fiber properties. At the molecular level, approximately 10% of the genome is linked to cell wall construction, dynamic architecture, and sensing functions (Carpita, 2011), and a major challenge is to identify which ones are involved in this process and to understand their roles. Globally, flax is a well-studied plant from a molecular genetics point of view – its genome has been sequenced (Wang et al., 2012; You and Fofana, 2023; You et al., 2023) and chromosome pseudomolecules have been assigned (You et al., 2018), an integrated consensus genetic and physical map was constructed (Cloutier et al., 2012), and the karyotypes of common flax (*Linum usitatissimum* L.) (Rachinskaya et al., 2011) and wild species were also investigated (Muravenko et al., 2001). Moreover, the flax plant is a convenient object to study cell wall development because the stem contains both cell walls rich in cellulose (bast fibers) and lignin (xylem cells) (Day et al., 2005; Gorshkova et al., 2012; Huis et al., 2012; Petrova et al., 2022). Several previous studies on flax using Omics together with biochemical and imaging approaches have identified changes in the expression of numerous genes (Hotte and Deyholos, 2008; Fenart et al., 2010; Huis et al., 2012; Zhang and Deyholos, 2016; Chabi et al., 2017; Gorshkov et al., 2017; Gorshkova et al., 2018; Simon et al., 2018; Gorshkova et al., 2023; Somalraju and Fofana, 2023). In many cases, the interpretation of the results was based on the hypothesis that changes in the expression of genes related to the biosynthesis of major cell wall polymers (e.g., cellulose, hemicellulose, pectins and lignin) could be related to differences in cell wall structure and/or fiber properties. However, while such an hypothesis is no doubt basically correct, it is also possible that changes in the expression of other, less obvious genes (i.e., not apparently related to cell wall metabolism) could affect key fiber parameters such as fineness and mechanical properties.

In an attempt to obtain further information about underlying molecular processes potentially related to flax fiber quality, we combined Omics and morphometric/mechanical analyses in bast fiber bearing tissues (a complex mixture of tissues that mainly contains bast fibers, but also epidermis, cortical parenchyma and phloem sieve tubes) of seven flax varieties belonging to three contrasted morphotypes: fiber spring (Belinka, Diane, Drakkar, Hermes), fiber winter (Adelie and Violin) and oil winter (Oliver) grown under field conditions. A panel of statistical analyses allowed us to highlight a limited number of relevant central actors of *in-planta* and *ex-planta* fiber quality. Their role in the context of elementary fiber development and fiber bundle quality is discussed.

## 2 Materials and methods

### 2.1 Plant growth and sample collection

The seven flax cultivars (Diane, Hermes, Drakkar, Belinka, Adelie, Violin, and Oliver) were grown under field conditions in plots at Terre de Lin (TdL), Saint-Pierre-le-Viger, France, except for the cultivar Violin, cultivated by LINEA, Grandviliers, France. All retting and scutching were performed by Terre de Lin. For Omics and morphometrics, plant samples were col2lected from three randomly distributed areas of the field at the budding stage. Five-cm-long fragments were removed from stems at 5 cm above the cotyledons and outer tissues (peeled off fiber-enriched outer part of stem) were manually separated from the inner tissues and frozen in liquid nitrogen before storing at -80° C until used for transcriptomic and proteomic analyses. The peeled off tissues are therefore a complex mixture of tissues that contain, besides bast fibers, at least epidermis, cortical parenchyma and phloem sieve tubes. A 1-cm long fragment was removed from the stem at 10 cm above the cotyledons from the same plants for morphometric analyses. For analyses of mechanical properties, plants were pulled (harvested) at maturity, field-retted, and scutched to produce fiber bundles corresponding to the length of the flax stems.

### 2.2 Morphometric analyses

Measurements were performed on one-cm stem fragments, collected 10 cm above the cotyledon from the same plants used for Omics approaches. Eighty-one fiber bundles [3 fiber bundles x 3 cross-sections x 3 individuals x 3 field areas] per cultivar were examined. Two hundred and seventy measurements [10 measures x 3 cross-sections x 3 individuals x 3 field areas] per variety were made to determine the fiber cell surface, including the G-layer of secondary cell wall also called tertiary cell wall (but excluding the common middle lamella with the adjacent fiber).

Preliminary observations were made on freehand cross-sections, without fixation in ethanol, and double stained with Alcian Blue (0.1%, w/v in water, 5 min), followed by Safranin O (0.1%, w/v in EtOH, 30 secs), mounted in water, and observed on an Olympus BH-2 light microscope. Remaining stem fragments were fixed in 80% ethanol, then dehydrated using a graded ethanol series. According to the manufacturer’s protocol, three individual stem fragments per field area were embedded in Technovit 7100 (Hereaus). Semi-thin sections (5 μm) were obtained with a Leica RM2065 microtome with glass knives. Three spatially separated cross-sections (every 200 μm) were selected for each individual for measurements. Toluidine-Blue O (TBO) coloration (0.1%, w/v in water) was applied on semi-thin sections to facilitate visual separation between the inner and outer tissues, allowing discrimination of fiber bundles and cell types. Measures were acquired with FIJI free software^1^. Cell surface and number of elementary fibers per bundle were further referred to as “*in-planta”* features.

### 2.3 Mechanical analyses of scutched fiber bundles

The following settings were used to characterize the tensile mechanical properties of flax fiber bundles from the 7 flax varieties (55 fiber bundles per variety were tested). The force-displacement curves were measured with Fafegraph M (Textechno GmbH, Mönchengladbach, DE; clamping length: 20 mm; test speed: 20 mm/min; pretension: 250 mg) (Haag and Müssig, 2016).

The thickness of the scutched fiber bundles of the 7 studied flax varieties were measured with at least 15 000 individual values by the scanner-based image analysis system FibreShape 5.1 (IST AG, Vilters, CH). The fiber bundles were cut to a length of approx. 20 mm and distributed on the scanner surface. The images were taken at a resolution of 2400 dpi (Epson Perfection V700, Epson GmbH, Meerbusch, DE). Six images per fiber batch were measured with the measuring mask Long_fibres_2400dpi_Epson_Bast_uncalib in the FibreShape software (minimum element width: 11 µm). The cross-sectional area was calculated from the thickness value of the fiber bundle using a SEM based correction model (Haag and Müssig, 2016):

Area = d^1.7256^ * 0.21113, where d – the thickness of the scutched fiber bundle.

Strength, Young’s modulus, strain, maximum force, and thickness measurements were further refered as “*ex-planta”* features.

### 2.4 Omics analyses

#### 2.4.1 Transcriptomics

##### 2.4.1.1 RNA extraction and quality verification

Total RNA was extracted from three pools of outer stem tissues for each of the 7 varieties using TRI-Reagent (MRC, Inc., Dundee, Scotland, UK). RNA integrity and concentration were evaluated with RNA StdSens Chips using the Experion automated electrophoresis system (Bio-Rad, Marnes-la-Coquette, France).

##### 2.4.1.2 Hybridization and data analyses

RNA processing, Cy3-labeling and hybridization were made following the manufacturer’s instruction for One-Color Microarray base gene Expression Analysis (Agilent Technologies). Hybridization was performed on Agilent microarrays Agilent-045382 UGSF flax 45K v1.0 array based upon flax genome coding sequence (*Lus-names*) (Wang et al., 2012) available at Phytozome^2^. Some genes that have genolin-names (Fenart et al., 2010) were added according to the EST database. The 4-plex array contains 45,220 60-mer *in situ* oligonucleotides per block. Hybridization and washing were performed following Agilent manufacturer’s instruction, and slides were immediately scanned at 5 mm pixel^-1^ resolution using an Axon GenePix 4000B scanner driven by GenePix Pro 6.0 software (Molecular Devices Corporation, Sunnyvale, CA, USA).

Scanned images (TIFF format) were then submitted to grid alignment and expression data analyses. For each slide, a lowest and print-tip median normalization was performed using R packages^3^ as implemented in CLC bio software^4^ followed by an inter-slide normalization. The 3 control samples were filtered for p-value < 0.05, and the average was calculated for each gene. A fold change (FC) value was calculated between individual treated samples and the mean of corresponding controls. Differentially expressed genes (DEGs) were selected for a threshold >2.0 or ≤0.5. Functional annotation of DEGs was based on NCBI GenBank, and related genes’ physiological processes were assigned with NCBI, AmiGO 2 Gene Ontology and UniProt. KEGG pathway analysis was also used to identify relevant biological pathways of selected genes.

#### 2.4.2 Proteomics

##### 2.4.2.1 Soluble protein extraction

Four grams of each of the three pools of frozen outer stem material were ground in liquid nitrogen to a fine powder, followed by 5 min grinding in 10 ml of Tris HCl buffer 50 mM, 0.06% IPC, pH 7.5, before centrifuging 10 min at 4°C/16,000 g. The supernatant was incubated for 15 min at room temperature with protamine sulfate under a low agitation, then centrifuged for 10 min at 18,000 g. The pellet was discarded, and the proteins were precipitated with 10% TCA (mass/v), 1 hour at -20°C. The pellet was washed once with cold acetone, and then dried for 5 min at room temperature (Goulas et al., 2001).

##### 2.4.2.2 Protein labeling

Dry protein pellets were resolved in 2D-DIGE labeling buffer (7 M urea, 2 M thiourea, 4% (mass/v) CHAPS and 30 mM Tris) at room temperature with agitation for 1 hours. The pH of the solution was adjusted to 8.5, and protein concentration was determined by 2D Quant kit (GE Healthcare). The protein samples were labeled with CyDye minimal dyes (GE Healthcare) according to the manufacturer’s instructions and as described in Grimaud et al. (2013). 30 µg of proteins were labeled by adding 240 pmol of fluorochromes (Cy3 or Cy5). A pooled internal standard was performed by mixing 15 µg of each sample and labeled with Cy2 dye and included in all gel runs (Goulas et al., 2006).

##### 2.4.2.3 Electrophoresis

For each 2-D gel a mixture with a final volume of 120 µl containing two samples labeled Cy3 and Cy5, internal standard labeled with Cy2, and labeling buffer [7 M urea, 2 M thiourea, 4% (mass/v) CHAPS, 6 µl.ml^−1^ DeStreak reagent (GE Healthcare)] and 1.5% (v/v) Biolyte, were loaded onto a 24 cm IPG non-linear strip with a pH range 3−10 (Bio-Rad) and separated using the IPGphor3 system with a rapid voltage slope to reach the maximum of 10 000 V and a total of 72 000 Vh. Second dimension resolution was carried out by SDS-PAGE on a 12.5% (v/v) resolving gel (HPE, Gel company) according to 2D HPE Large Gels manufacturer’s instructions.

##### 2.4.2.4 Image analysis and protein identifications

Scanning of the 2D-DIGE gels was carried out using a Typhoon Imager 9400 (GE Healthcare) at three different wavelengths corresponding to the different CyDyes as described in Grimaud et al. (2013). The generated gel images were analyzed with DeCyder 7.0 software, using the Cy2 channel as a standard. The multiple 2-D DIGE gels were matched, and the significance of changes in the abundance of specific proteins from different genotypes was established. By including the internal standard on each gel in the experiment, together with the individual biological samples, it is possible to measure the abundance of each protein spot on a gel relative to its corresponding spot in the internal standard present on the same gel (i.e. as a ratio). All matched spots were manually verified on all gels.

##### 2.4.2.5 Enzymatic digestion and protein identification

Differentially abundant spots (based on the statistical analyses described below) were excised from the gels and digested using an EVO2 Workstation (TECAN). All MS and MS/MS analyses were carried out by using a 5800 MALDI TOF/TOF (AB Sciex, Foster City CA, USA) (Chabi et al., 2017), and Protein identification was performed using MASCOT server with a *Linum* database downloaded from phytozome (Lusitatissimum_200_V1.0 with 43484 sequences; 17356610 residues) according to (Wang et al., 2012).

### 2.5 Bioinformatics and statistical analyses

#### 2.5.1 Data deposition

Transcriptomic data have been deposited to the NCBI online repository with accession number GSE222066. The mass spectrometry proteomics data have been deposited to the ProteomeXchange Consortium via the PRIDE^5^ partner repository (Perez-Riverol et al., 2022) with the dataset identifier PXD039389.

#### 2.5.2 Transcriptomic analysis

A script utilizing library functions in R with a Bonferroni-corrected p-value of less than 0.05 and a cut-off of +/- 1.5 log2 ratio was used for all experimental conditions to identify genes displaying a significant change in expression (differently expressed gene DEG) over the repetitions. Only genes with smooth expression profiles were retained. Differential analyses were performed for each variety vs the other 6 varieties giving 21 possible combinations. The differentially expressed genes and differently abundant proteins were annotated according to KEGG database using ShinyGO online tool^6^ (Ge et al., 2020). The MapMan framework for functional classification (Schwacke et al., 2019) with Mercator4 v.2 online tool^7^ was also used to assign the protein sequence annotation.

#### 2.5.3 Proteomic analysis

Statistical analyses were performed with the Extended Data Analysis (EDA) module in DeCyder 2D Differential Analysis Software v.7.0 (Goulas et al., 2006). For every set of matched spots, the average intensity was compared to the standard deviation on these intensities for a given spot between the two compared varieties. Only spots present on all gel images from each set of samples were considered. One-way ANOVA analysis was performed for every set of matched spots, comparing the average and standard deviation for a given spot between Diane and Oliver groups to find significant differences (ANOVA, p-value ≤ 0.05; average ratio >1.5) above the experimental variation.

#### 2.5.4 Global statistical analyses

Sparse Partial Least-Squares Discriminant Analysis (sPLS-DA) analyses were conducted by using RStudio software (2022.02.03 build 492) and the R-package mixOmics (v. 6.1.2). The PLS method searches for the highest covariance between variates, which are a linear combination of initial variables, taking into account all variables in the input matrices. sPLS combines this approach with a Lasso penalization to enable the selection of variables the most explanatory of varieties discrepancy. The arithmetic means of the gene expression value and their log2 transformation were used for statistical analysis. Morphometric and mechanical data were tested for normality by the Shapiro-Wilk test. We used the ANOVA test, Pearson correlation coefficient, Student’s t-test for normally distributed data, Spearman correlation, and the Kruskal-Wallis test if the normality test was not met. We used the R lm-function to generate the regression plots. The calculated determination coefficient (R^2^) was used to estimate the proportion of variation of the dependent variance that could be explained by the variation of the independent variance.

## 3 Results

### 3.1 Morphometric characterization of the 7 varieties, in-planta features

**Figure 1:**
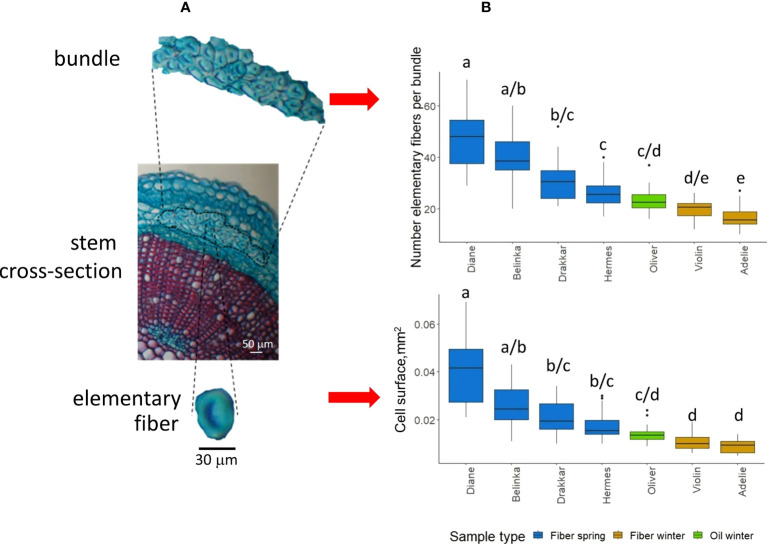
Flax fiber morphometric properties. **(A)** Light microscope image showing flax stem cross-section (middle), fiber bundle (top), and a single fiber cell (bottom). Sample stained in safranin O/alcian blue. **(B)** Box-plot of two morphometric features, the number of elementary fibers per bundle (upper), and the cell surface of single fibers within fiber bundles (lower) for the seven flax varieties (Diane, Hermes, Drakkar, Belinka, Adelie, Violin, and Oliver). Letters shows statistical differences between flax varieties, p-value ≤ 0.05 (Dunn’s test).

We determined the number of elementary fiber cells per bundle and fiber surface in four flax spring fiber varieties (Belinka, Diane, Drakkar, Hermes), two winter fiber varieties (Adelie, Violin) and one winter oil variety (Oliver) (**Figure 1**). The results show a large scale of variation for both parameters with all fiber spring varieties showing the highest values and fiber winter varieties the lowest. The oil variety Oliver showed intermediate values.

### 3.2 Transcriptomic analysis of 7 flax varieties and correlation with morphometric parameters

**Figure 2:**
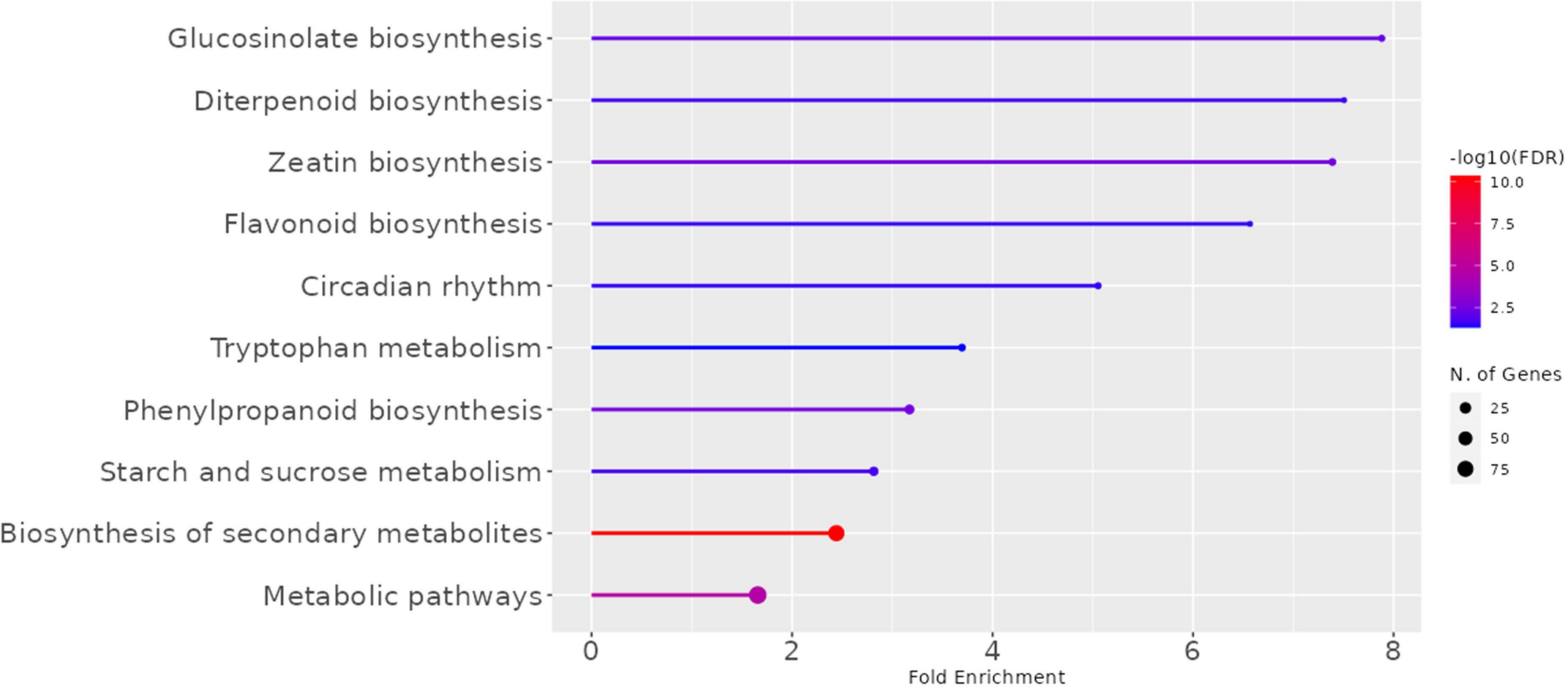
KEGG Pathways enriched in the transcriptome of fiber-bearing outer-stem tissues. The 1041 DEGs of the 7 varieties (Diane, Hermes, Drakkar, Belinka, Adelie, Violin, and Oliver) were selected, and their *Arabidopsis thaliana* orthologues were compared to the gene list of the complete *Arabidopsis* genome. Line length corresponds to the fold of enrichment. The Line color indicates the FDR value. The size of the end spot shows the number of genes from the 1041 *LusID* DEGs list that are identified in the corresponding *A. thaliana* KEGG-category.^10^

We evaluated the abundance of 42 963 transcripts (99.3%) among the 43 384 annotated genes in the flax genome^8^. Comparative whole transcriptomics of the fiber-bearing outer-stem tissues from the 7 varieties allowed us to identify 1041 differently expressed genes (DEGs) that showed significant differences in transcript accumulation (**Figure 2**; **Supplementary Table 1**). According to MapMan annotation, only 40 of them were classified into cell wall organization (**Supplementary Table 1**, bold characters). However, further expert curation allowed us to highlight 57 more genes related to cell wall metabolism (**Supplementary Table 2**). These 1041 DEGs were also annotated according to KEGG categories (**Figure 2**). Our results show that glucosinolate-, diterpenoid- and zeatin-biosynthesis are pathways in which the genes are the most overrepresented (up to 8-fold). Circadian rhythm, tryptophan, starch, sucrose metabolism, and phenyl biosynthesis also showed significant enrichment (approx. 4-fold). Finally, biosynthesis of secondary metabolites and metabolic pathways showed less fold-enrichment (approx. 2-fold), but with a much higher number of significant DEGs.

**Figure 3:**
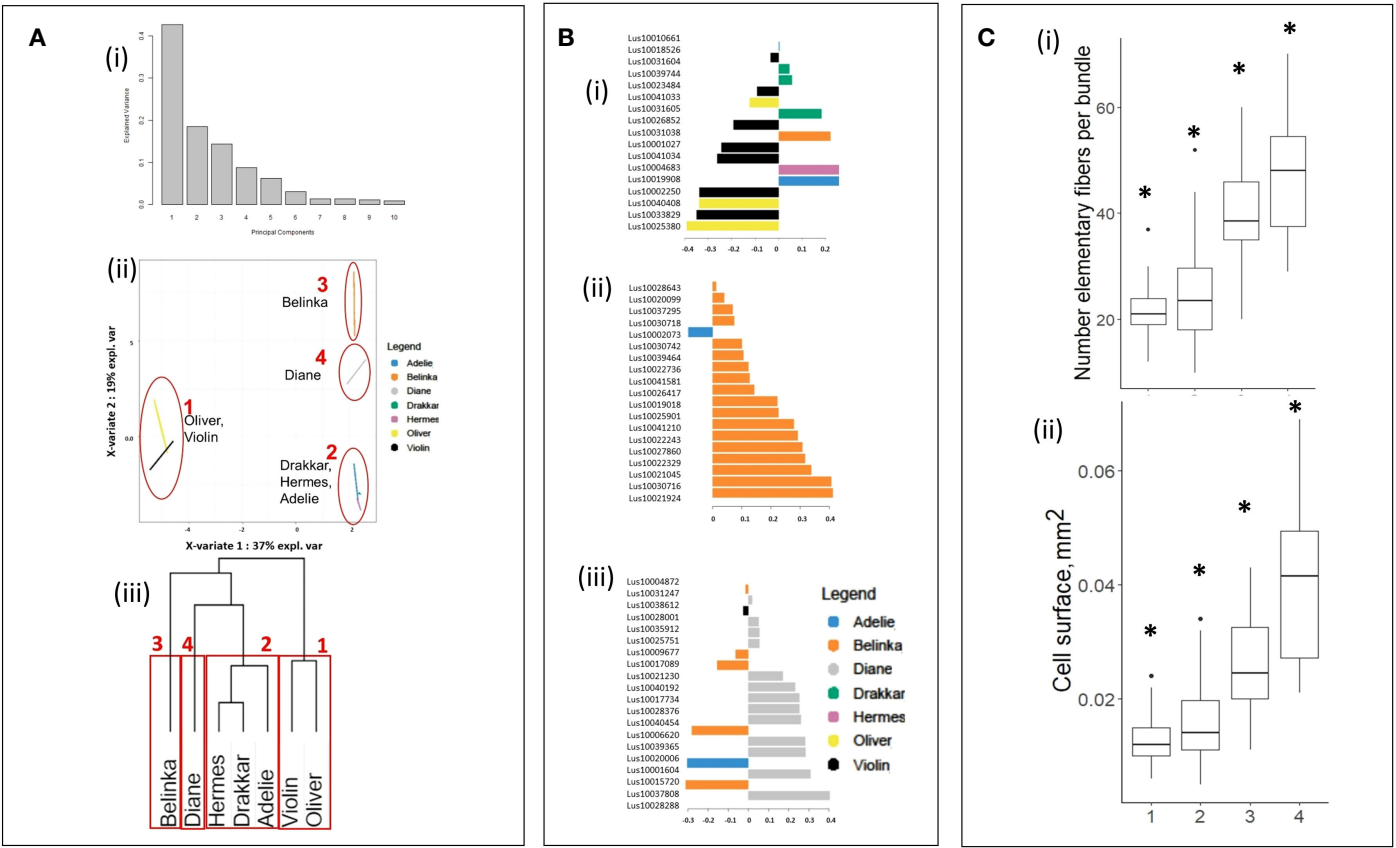
Statistical clusterization of the 7 flax varieties based upon DEG values from transcriptomic analysis. **(A)** (i) Scree plot explained variance of PCA, (ii) plot of sparse Partial Least-Squares Discriminant Analysis (sPLS-DA), and (iii) hierarchical clusterization on Euclidian distance^11^, **(B)** Contribution plots of the DEGs on X-variates 1 (i), 2 (iii) and 3 (iii) of sPLS-DA, **(C)** Boxplots of (i) number of single fiber cells and (ii) cell surface of fiber cells within clusters highlighted in **(A)**. Asterisks indicate statistically significant differences (p-value ≤ 0.05) between groups..

All 1041 significant DEGs were processed with mixOmics R-package to Principal Component Analysis (PCA, **Figure 3A**-i), and Sparse Partial Least-Squares Discriminant Analysis (sPLS-DA, **Figure 3A**-ii). As shown in **Figure 3A**-i, the PCA scree plot showed that 75% of the total variance was explained with the first three principal components (43, 18, and 14% of the total variation, respectively). Thus, the 3 principal components were selected to further sPLS-DA (**Figure 3A**-ii) which showed that 73% of the total variance was explained by the first three X-variates (37, 19 and 17%, respectively), corresponding to 56 DEGs. When considering the X-variate 1, a clear discrimination was noticed between the first cluster, composed of the two winter varieties, one of them is fiber flax (Violin), and the other one is oil (Oliver), and all the other varieties. The X-variate 2 showed discrimination between this first cluster and a second one composed of two spring (Drakkar, Hermes) and one winter (Adelie) fiber varieties, a third composed of one spring fiber variety (Belinka), and a fourth one composed of another spring fiber variety (Diane). Such clusterization was further confirmed by hierarchical clusterization on Euclidian distance (**Figure 3A**-iii).

The contribution of each variable as a component of sPLS-DA indicate the importance of that variable for discrimination. We identified a total of 56 DEGs transcripts that have higher contribution values than others of the studied transcripts (**Supplementary Table 3**), 17 transcripts for X-variate 1 (**Figure 3B**-i), 19 transcripts for X-variate 2 (**Figure 3B**-ii), and 20 transcripts for X-variate 3 (**Figure 3B**iii; **Supplementary Table 3**). The DEGs that were the most explanatory for sPLS-DA X-variate 1 were down-regulated only in Oliver and Violin, whereas others were up-regulated in Drakkar, Belinka, Hermes, and Adelie. For X-variate 2, the explanatory DEGs are almost all up-regulated in Belinka only, with the exception of a single down-regulated gene in Adelie. For X-variate 3, the most explanatory DEGs were down-regulated, especially in Belinka, or up-regulated only in Diane.

The variation of morphometric parameters was calculated within each of the four clusters (**Figure 3C**) highlighted after analysis of the 1041 DEGs (**Figures 3A**ii, iii). Based on the transcriptome analysis, we discriminate groups of varieties that differ significantly in morphometric traits. They showed significant differences in the studied phenotypic traits between the four clusters. The varieties within the 4th cluster have more individual fibers in bundles, and their cell diameter was the highest. And *vice versa*, the varieties joined in the first cluster carried fewer single cells in fiber bundles, and the cells were smaller.

**Figure 4:**
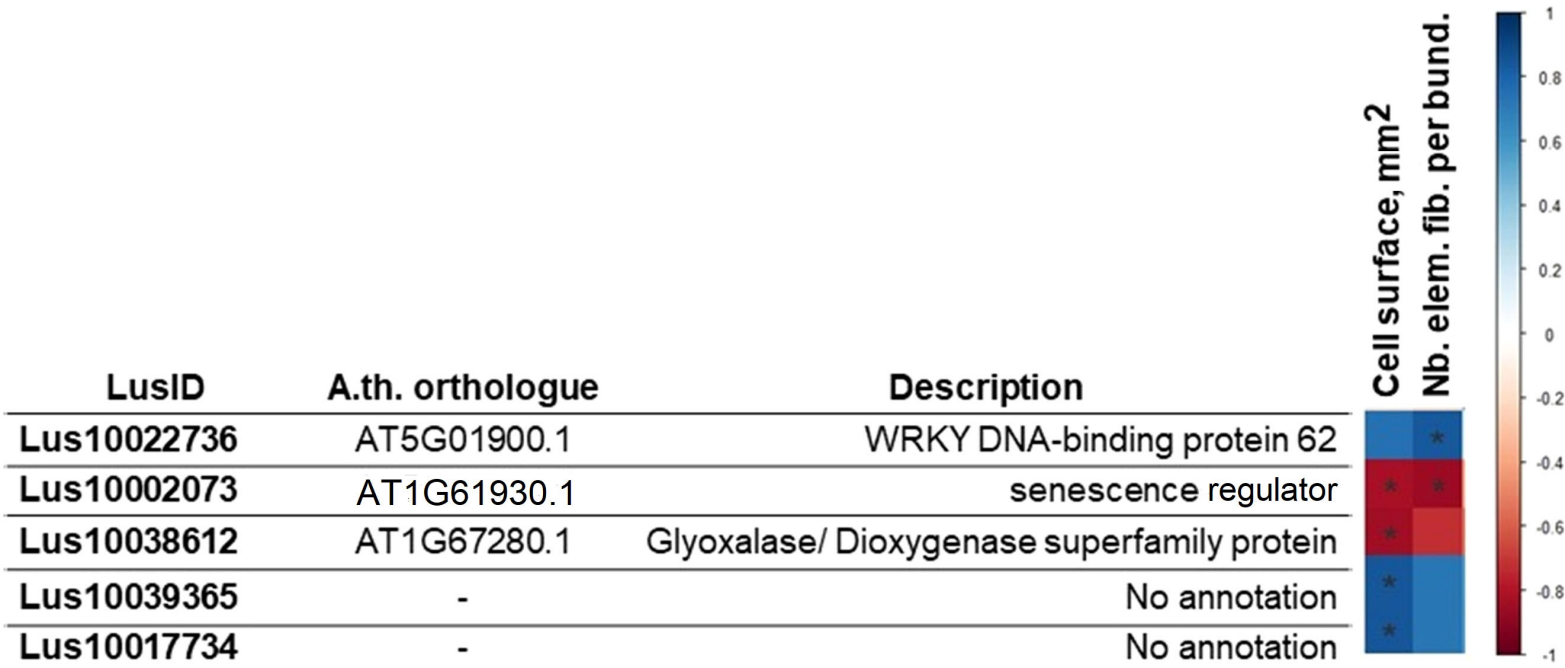
Pearson correlation coefficient (PC) between the expression level of genes selected on transcriptomic data analysis and morphometric (the surface of a fiber cell, mm^2^; the number of fibers in the bundle). The p-value for each PC was calculated, and the PC with p-value ≤ 0.05 are given in the table. The red cell color marks a negative correlation, the blue one a positive one. * - p-value of PC ≤ 0.05.

The Pearson correlation coefficient between the expression level of the 56 genes highlighted after sPLS-DA analysis of bast fiber transcriptome and values of morphometric features (the number of elementary fiber cells in the bundle and the single cell surface) of the same varieties were calculated (**Figure 4**). We identified 5 genes whose expression level had a strong statistically significant correlation with studied morphometric features (maximum value 0.84; minimum value -0.86) (**Figure 4**). One gene was identified as the *LusWRKY85* (*Lus10022736*) gene, which is a transcription factor of the WRKY family, and it is orthologous to the *Arabidopsis AtWRKY62 (AT5G01900.1)* (Yuan et al., 2021). Another gene is *LusS40-6 (Lus10002073)*, and its *Arabidopsis*’s orthologue *AtS40-6* (*AT1G61930*) codes senescence regulator (Fischer-Kilbienski et al., 2010). The next gene in the list is *LusGLYI6* (*Lus10038612*), and it is also negatively correlated with cell surface (**Figure 4**). The *Lus10038612* gene has homology with *AtGLYI6 (AT1G67280.1)*, which encodes a Ni^2+^-dependent glyoxylase (Batth et al., 2020). Finally, two genes (*Lus10039365* and *Lus10017734*) with unknown functions were also highlighted (**Figure 4**).

### 3.3 Proteomic analysis of bast fibers from a winter oil and a spring fiber variety and correlation with morphometric parameters

A comparative proteomic analysis was performed on the peeled off fiber-enriched outer stem parts of Diane and Oliver since these varieties belonged to the two transcriptomics clusters showing the most discriminant and significant differences regarding morphometric measurements (**Figure 3**). A total of 1490 protein spots were identified on the 2D gel. Among these, 108 showed significant differential abundance (p.value ≤ 0.05) with at least a 1.5-fold change between the two varieties (7.25% of protein spots in the gel). Subsequent excision, trypsin digestion, and mass spectrometry allowed the successful identification of 46 proteins representing 32 non-redundant proteins (**Table 1**), among which 15 of them were more abundant in Diane and 17 in Oliver (**Supplementary Figure 1**). One peptide spot (Master number 1046) that was more abundant in the Diane proteome represents a mixed sample, which included two peptides identified like ascorbate peroxidase 1 and ferretin 1 (**Table 1**; **Supplementary Figure 1**). Both of them were included in further analysis. The transcript annotations of genes coding the 32 identified proteins were classified according to MapMan annotation, and only 1 of them could be classified directly into cell wall organization, *Lus10037377*, a xyloglucan endotransglucosylase/hydrolase 32 [homologous to ELF4-like in *Arabidopsis thaliana* genome (*AT2G36870.1*)] (**Supplementary Tables 1, 2**).

**Table 1:** Proteins and their gene IDs differently abundant in Diane and Oliver flax varieties.

N	Transcript	Abundance ratio	P-value	Master numb.	Arabidopsis ortholog	Gene abbreviation	Pfam domen(s)	Definition according to *At* genome
			*Proteins (and their genes) that are more abundant in Diane*					
1	Lus10008932	4.07	2.6E-05	1269	AT4G14060.1		PF00407	Polyketide cyclase/dehydrase and lipid transport superfamily protein
2	Lus10009205	2.54	5.2E-05	953	AT2G23580.1	ABE4, ATMES4, MES4		methyl esterase 4
3	Lus10037377	2.38	1.2E-02	859	AT2G36870.1	XTH32	PF00722, PF06955	xyloglucan endotransglucosylase/hydrolase 32
4	Lus10010846	2.09	6.4E-04	890	AT2G37660.1		PF01073	NAD(P)-binding Rossmann-fold superfamily protein
5	Lus10023569	2.03	1.5E-03	1350	AT2G21660.1	ATGRP7, CCR2, GR-RBP7, GRP7	PF00076	cold, circadian rhythm, and rna binding 2
6	Lus10015049	1.99	2.8E-06	941	AT5G02790.1	GSTL3	PF00043	Glutathione S-transferase family protein
7	Lus10041602	1.75	1.1E-02	1125	AT3G52150.1		PF00076	RNA-binding (RRM/RBD/RNP motifs) family protein
8	Lus10023180	1.67	3.6E-06	1307	AT1G65980.1	TPX1	PF08534	thioredoxin-dependent peroxidase 1
9	Lus10026241	1.63	7.2E-03	797	AT5G36700.1	ATPGLP1, PGLP1		2-phosphoglycolate phosphatase 1
10	Lus10010340	1.58	4.8E-06	639	AT1G04420.1		PF00248	NAD(P)-linked oxidoreductase superfamily protein
11	Lus10031708	1.56	4.3E-05	183	AT3G60750.1		PF02779, PF02780, PF00456	Transketolase
12	Lus10013537/ Lus10012499, Lus10022673	1.55	4.7E-03	1046	AT1G07890.1/AT5G01600.1	APX1, ATAPX01, ATAPX1, CS1, EE6/ATFER1,FER1	PF00141/ PF00210	ascorbate peroxidase 1/ ferretin 1
13	Lus10017158	1.53	1.3E-02	1354	AT5G57040.1		PF00903	Lactoylglutathione lyase/glyoxalase I family protein
14	Lus10024383	1.50	3.2E-02	870	AT2G37660.1		PF01073	NAD(P)-binding Rossmann-fold superfamily protein
			*Proteins (and their genes) that are more abundant in Oliver*					
1	Lus10008932	9.78	1.0E-05	1284	AT4G14060.1		PF00407	Polyketide cyclase/dehydrase and lipid transport superfamily protein
2	Lus10014003	5.81	5.0E-06	1449				
3	Lus10012167	4.52	2.6E-05	1227	AT2G16600.1	ROC3	PF00160	rotamase CYP 3
4	Lus10030577	4.03	1.5E-04	198	AT5G24550.1	BGLU32	PF00232	beta glucosidase 32
5	Lus10001508	3.63	6.1E-06	878	AT4G34180.1		PF04199	Cyclase family protein
6	gi|171462119	1.80	3.4E-04	1420				Expansin-related protein 3 precursor
7	Lus10043025	1.77	6.6E-04	249	AT5G25880.1	ATNADP-ME3, NADP-ME3	PF00390, PF03949	NADP-malic enzyme 3
8	Lus10042468	1.66	4.7E-06	1078	AT1G78380.1	ATGSTU19, GST8, GSTU19	PF02798, PF00043	glutathione S-transferase TAU 19
9	Lus10025889	1.60	5.3E-03	1391	AT5G48580.1	FKBP15-2	PF00254	FK506- and rapamycin-binding protein 15 kD-2
10	Lus10013078	1.58	4.9E-03	1526	AT3G27830.1	RPL12, RPL12-A	PF00542	ribosomal protein L12-A
11	Lus10026370	1.54	2.9E-02	223	AT3G08590.1		PF01676, PF06415	Phosphoglycerate mutase, 2,3-bisphosphoglycerate-independent
12	Lus10035263	1.52	3.0E-02	402	AT5G08680.1		PF00006, PF11421, PF02874, PF00306	ATP synthase alpha/beta family protein
13	Lus10011125	1.51	5.4E-04	232	AT5G25880.1	ATNADP-ME3, NADP-ME3	PF00390, PF03949	NADP-malic enzyme 3
14	Lus10020717	1.51	1.6E-02	1165	AT1G06680.1	OE23, OEE2, PSBP-1, PSII-P	PF01789	photosystem II subunit P-1
15	Lus10002620	1.50	7.1E-03	69	AT5G17920.1	ATCIMS, ATMETS, ATMS1	PF01717, PF08267	Cobalamin-independent synthase family protein
16	Lus10018156	1.50	1.0E-04	291	AT1G21750.1	ATPDI5, ATPDIL1-1, PDI5, PDIL1-1	PF00085	PDI-like 1-1
17	Lus10026643	1.50	3.5E-04	1094	AT2G47730.1	ATGSTF5, ATGSTF8, GST6, GSTF8	PF02798, PF00043	glutathione S-transferase phi 8

*Arabidopsis* orthologs of the *Lus* DEGs of the 32 differently abundant proteins are presented as well as gene abbreviation and Pfam domains.

Interestingly, both in Diane and in Oliver, proteins with highest variations in abundancy ratio (**Table 1**) were two different protein spots (Master number 1269 and 1284, respectively) identified as the same polyketide cyclase/dehydrase and lipid transport superfamily protein (Lus10008932), roughly twice less in Diane (4.07) than in Oliver (9.78). This fact may indicate an alternative modification of the Lus10008932 protein in the proteome of two studied flax varieties. Such 2-fold range differences in ratio abundancy were also observed for a methyl esterase 4 (Lus10009205), a xyloglucan endotransglucosylase/hydrolase 32 (Lus10037377), a NAD(P)-binding Rossmann-fold superfamily protein (Lus10010846) and a cold, circadian rhythm, and RNA binding 2 protein (Lus10023569) whose ratio variation was around 2.3 in Diane whereas a unassigned protein (Lus10014003), a rotamase CYP 3 (Lus10012167), a beta glucosidase 32 (Lus10030577) and a cyclase family protein (Lus10001508) showed a ratio variation of around 4.5 in Diane. Nine proteins in Diane and twelve in Oliver showed variation in abundance ratio of roughly 1.5. It has to be noticed that the four proteins directly or indirectly linked to cell wall development were differently abundant in the studied varieties. Two of them (Lus10009205 – methyl esterase 4 and Lus10037377 – xyloglucan endotransglucosylase/hydrolase 32) were more abundant in Diane (2.54 and 2.38 times, respectively), and the other two (Lus10030577 – beta glucosidase 32 and gi|171462119 – Expansine-related protein 3 precursor) – in Oliver (4.03 and 1.80 times, respectively) (**Table 1**).

**Figure 5:**
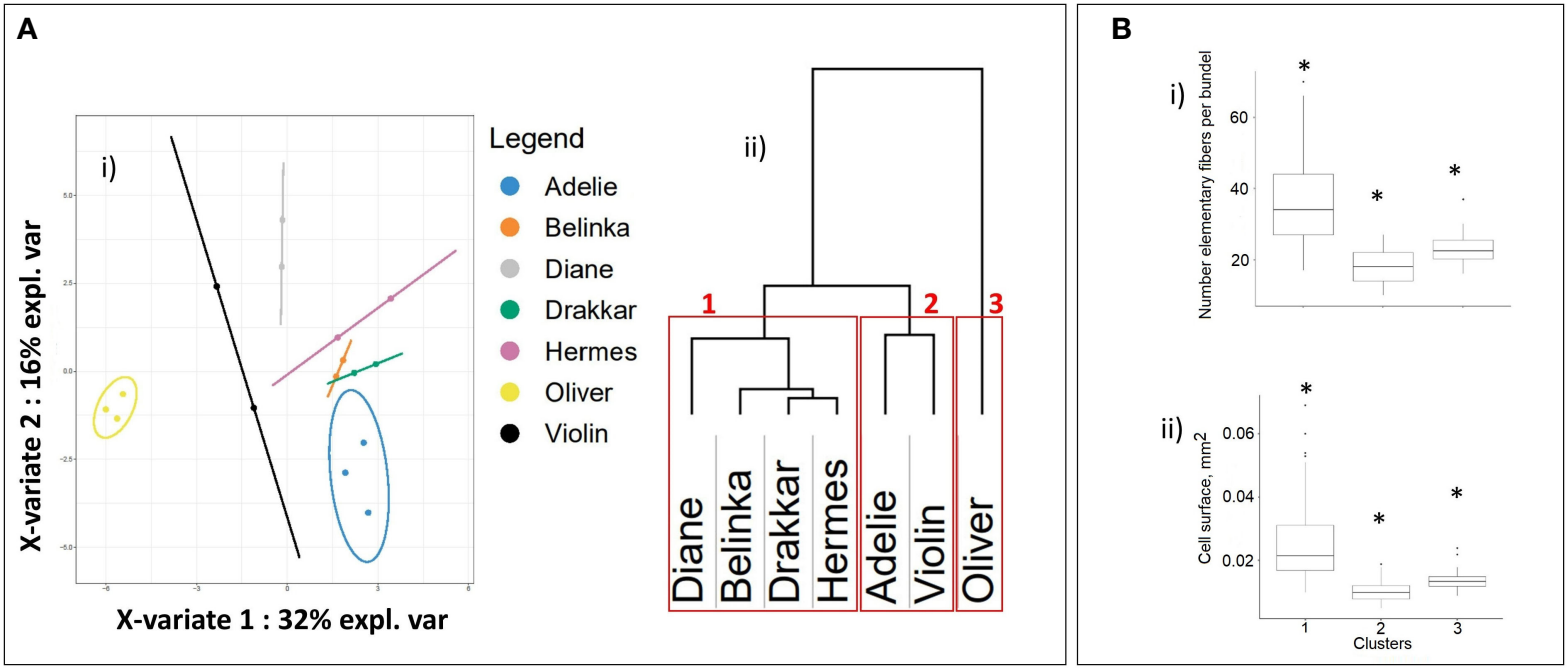
Statistical clusterization of the 7 flax varieties based upon expression levels of 32 genes corresponding to non-redundant proteins selected after statistical proteomic analysis. **(A)** (i) Partial Least-Squares Discriminant Analysis (PLS-DA), (ii) hierarchical dendrogram^12^, the red frames show three groups of flax varieties that corresponded to spring fiber (1), winter fiber (2), and winter oil (3) varieties. **(B)** Boxplots of (i) individual fiber cells and (ii) surface of fiber cells within clusters highlighted in Figure 5A after conversion of the 32 candidate proteins protein IDs to gene *LusIDs* and then gene sequences. Asterisks indicate statistically significant differences (p-value ≤ 0.05) between groups.

Since proteomic data was only available for two varieties it was not possible to evaluate the relative weight of the 32 identified proteins as potential markers for fiber-related parameters in the complete panel of 7 varieties. The expression profiles of the genes corresponding to the 32 candidate proteins IDs to gene IDs were therefore further established for the 7 flax varieties studied. Clusterization based on their expression level showed clear discrimination into three separate clusters (**Figure 5A**). The first cluster differentiated spring fiber varieties Diane, Belinka, Drakkar, and Hermes from winter fiber varieties Adelie and Violin (the second cluster). The winter oil flax Oliver formed the most distant cluster (the third one) from the other flax varieties. It should be noted that the cluster of the spring fiber varieties combines samples with the highest amount of fiber cells in the bundle and the biggest cell surface (**Figure 5B**).

**Figure 6:**
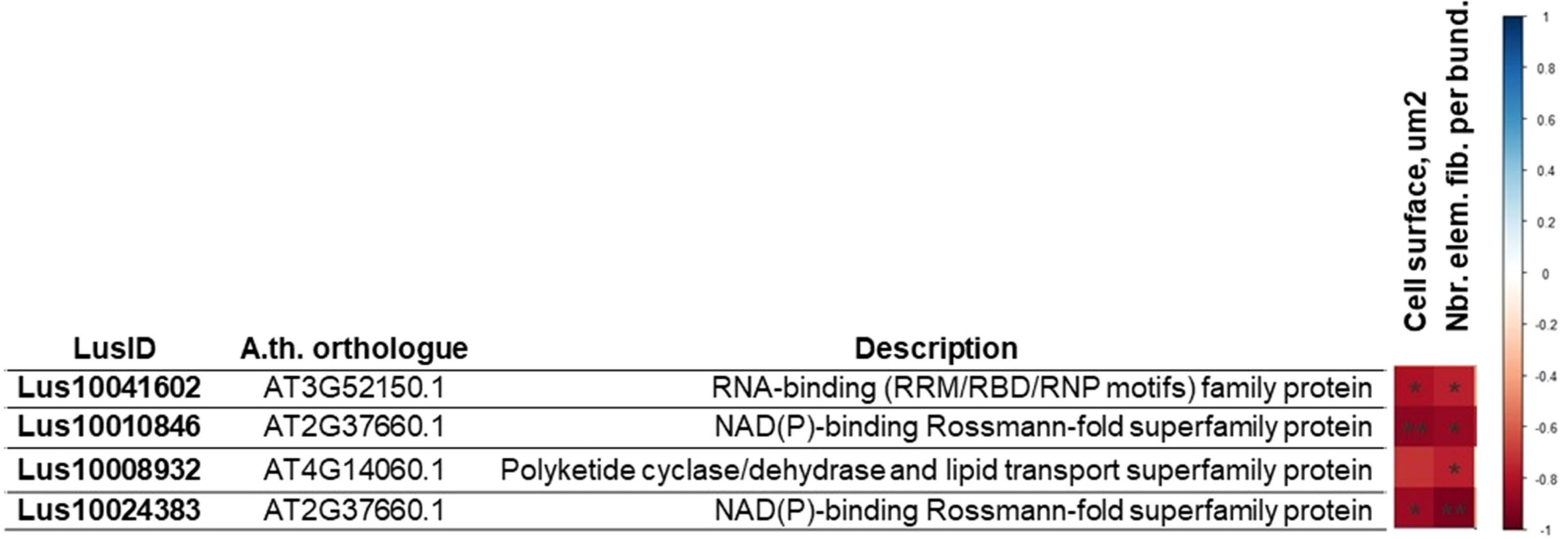
Pearson correlation coefficient (PC) between the expression level of genes selected on proteomic data analysis and morphometric (the surface of a fiber cell, mm^2^; the number of fibers in the bundle). The p-value for each PC was calculated, and PC with p-value ≤ 0.05 are given in the table. The red cell color marks a negative correlation, * - p-value of PC ≤ 0.05, ** - p-value of PC ≤ 0.01.

Pearson correlation coefficients were further calculated between the expression levels of 32 genes based on the results of the proteomic study and the morphometric features of the same varieties (**Figure 6**). We identified 4 genes whose expression level strongly correlated with studied morphometric features (maximum value -0.72; minimum value -0.93) (**Figure 6**). Two genes (*Lus10010846* and *Lus10024383*) encoding putative 3-beta-hydroxysteroid dehydratase/isomerases were identified as putative lipid-binding proteins in phloem exudates of *Arabidopsis* plants (Guelette et al., 2012). Another gene (*Lus10008932*) potentially related to lipid metabolism was also identified (**Figure 6**); its product is described as a plant-specific protein (Gutierrez et al., 2004).

### 3.4 *Ex-planta* mechanical features of scutched fibers and correlation with gene candidates and in- planta morphometric parameters

**Figure 7:**
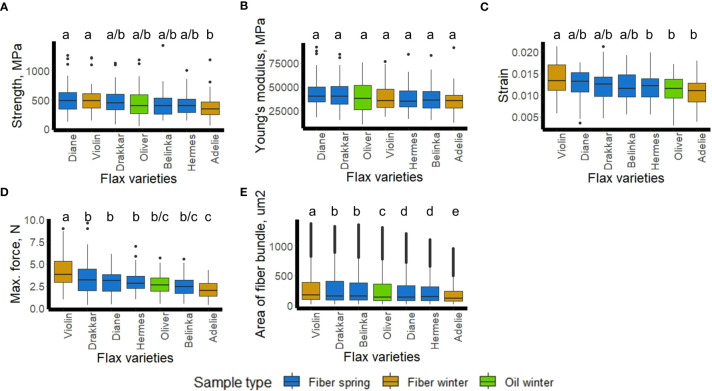
Flax fiber mechanical properties (**A**-Strength, **B**-Young’s modulus, **C**-Strain, **D**-Maximum force) and geometric properties (**E**-Area of scotched fiber bundles) of the 7 flax varieties (Diane, Hermes, Drakkar, Belinka, Adelie, Violin, and Oliver). Letters shows statistical differences between flax varieties, p-value ≤ 0.05 (Tukey’s test).

Mechanical (strength, Young’s modulus, strain, maximum force) and geometric (thickness) measurements, *ex-planta* features, were made on scutched fiber bundles obtained from the 7 varieties (**Supplementary Table 4**). Statistically significant differences for strength, strain, and maximum force were found at 95% of confident interval (**Figures 7A, C, D**), as for the thickness of the scutched fiber bundle (**Figure 7E**). We noticed significant differences of Young’s modulus between the seven varieties when we accepted 90% level of the confidence interval (not at 95%). The scattering of mechanical values is a common observation with plant fibers and it is related to the variability of these natural material. There is indeed a large range of factors which can affect variability, from plant growth conditions to fiber sampling, processing and testing (Baley et al., 2020). The average calculated values for each variety are in accordance with the results reported previously (Bourmaud et al., 2018).

We combined the two lists of candidate genes that we identified according to previous transcriptomic (**Supplementary Table 3**) and proteomic (**Table 1**) analyses to calculate Pearson correlation coefficients between expression levels and mechanical values measured for the same varieties. We identified 3 genes whose expression level was strongly correlated with mechanical properties (**Table 2**). One of the candidate genes is an expansin-like protein (*gi|171462119*) which is connected to cell wall metabolism (Roach and Deyholos, 2008). The two other genes (*Lus10011125* and *Lus10010846*) encode an enzyme that catalyzes the oxidative decarboxylation of L-malate, the NADP-malic enzyme 3, and an NAD(P)-binding Rossmann-fold superfamily protein, also identified as a putative 3-β-hydroxysteroid dehydratase/isomerase in *Arabidopsis* phloem exudates (Guelette et al., 2012).

**Table 2:** Pearson correlation coefficient (PC) between the expression level of selected genes and mechanical features (strength, Young’s modulus, strain, maximum force, and area of a fiber bundle).

LusID	*A. thaliana* orthologue	Description	Strength, MPa	Young’s modulus, MPa	Strain	Max. force, N	Area, mm^2^
Lus10011125	AT5G25880.1	NADP-malic enzyme 3	0.75*	0.45	0.56	0.59	0.61
Lus10010846	AT2G37660.1	NAD(P)-binding Rossmann-fold superfamily protein	-0.80*	-0.72	-0.51	-0.25	-0.38
gi|171462119		Expansin-like protein	0.28	-0.07	0.52	0.78*	0.54

The p-value for each PC was calculated, and PC with p-value ≤ 0.05 are given in the table. Red highlighting indicates a high correlation, * - p-value of PC ≤0.05.

**Figure 8:**
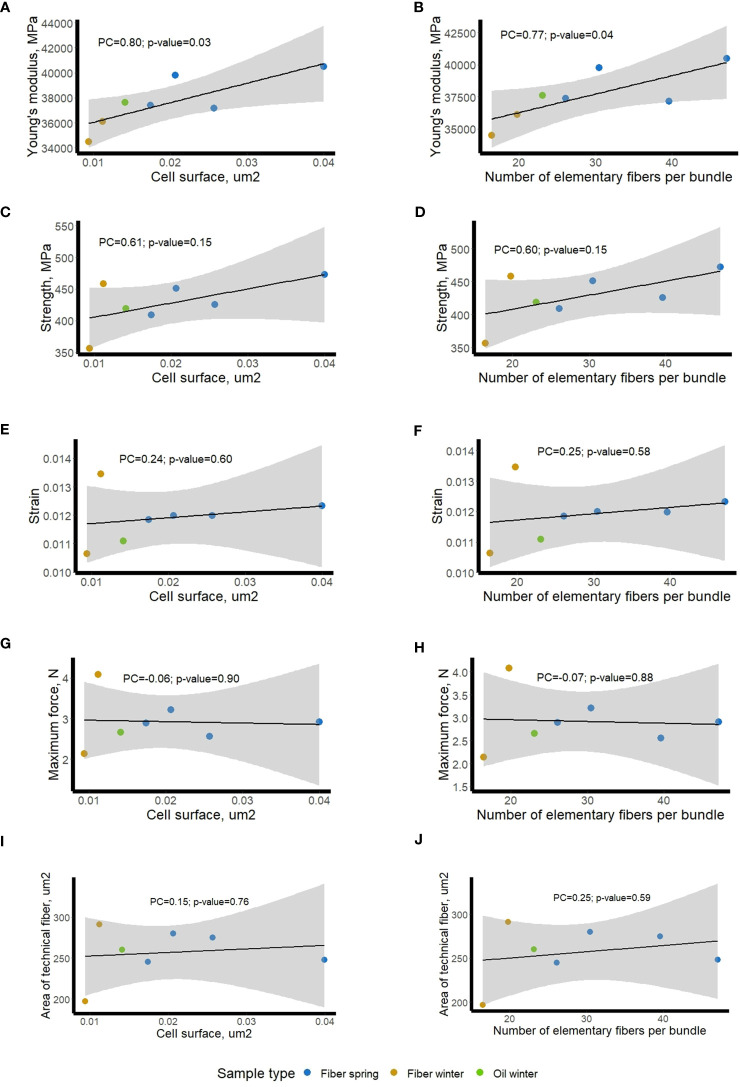
Relationship between mechanical data (*ex-planta*) of fiber bundles and morphometric data (*in-planta*) of stem fibers/fiber bundles in different flax varieties. Blue dots – fiber spring, bronze dots – fiber winter, and green dot – oil winter; mechanical data *vs* cell surface **(A, C, E, G, I)**; mechanical data vs bundle fiber number **(B, D, F, H, J)**. The regression line is plotted with a black line, a grey shaded area shows 95% confidence interval. Pearson Correlation coefficients (PC) and their p-values are given on the plot.

To see whether those *ex-planta* mechanical values (**Figure 7**) of scutched fibers could be related to *in-planta* fiber morphometric data (**Figure 1**), we calculated Pearson correlation coefficients with cell surface and number of fibers within bundles. The bivariate correlations between the 2 sets of morphological data (**Figure 1**) and the 5 sets of mechanical data (**Figure 8**) are shown on regression plots (**Figure 8**). The regression lines reflect positive correlations between both morphometric features and Young’s modulus (PC of 0.8 and 0.77, respectively) and both morphometric features and strength (PC of 0.61 and 0.60, respectively), whereas no correlation could be observed with strain, maximum force or with the area of scutched fibers. We also did not find any significant correlation between fiber bundle surface *in-planta* (calculated as the multiplication of cell surface and number elementary fibers per bundle) and scutched fiber area *ex-planta* (data not shown).

## 4 Discussion

The fineness and mechanical properties of fibers are key quality parameters for textiles and NFCs that are determined by two main processes in addition to scutching: i) fiber formation during the growth of the flax plant and ii) fiber separation and extraction during the post-harvest retting process (Djemiel et al., 2020). Both of these processes are, in turn, determined by the flax genotype and environmental conditions (including the composition of the retting microbiome). In this paper, we combined Omics performed on bast fiber bearing tissues and morphometric/mechanical analyses in seven flax varieties grown under field conditions to provide further information about putative key molecular actors of elementary (single) fiber constitution and fiber bundle properties.

The morphometric data revealed a relatively high degree of phenotypical variability between the different replicates for the seven varieties analyzed, most likely reflecting the combined effects of genetic and environmental factors as shown in previous studies (Stenström et al., 2002; Clarkson, 2005; Richter et al., 2012). Indeed, clear differences were observed both for elementary fiber number per bundle and fiber cross-sectional surface between the different varieties, with the highest values for the four spring fiber varieties and lowest for the two winter fiber varieties, the oil variety (Oliver) showing intermediate values. During retting and decortication process, fiber bundles are separated from each other but the individual fibers can still be linked within the bundles. The mechanical properties are dependent on the fineness of scutched fibers, i.e. the number of individual fibers in the bundles.

Comparative transcriptomics of fiber-enriched tissues from the seven varieties identified a total of 1041 DEGs of which 9.3% were related to cell wall metabolism (**Supplementary Tables 1, 2**). The majority of these genes is implicated in cell wall polymer modification including beta-glucanases, beta-galactosidases, glycosyl hydrolases, pectin lyase-like and xyloglucan endotransglucosylase/hydrolase. This is in the agreement with previous findings showing that the proteins identified in the outer stem tissues compared to inner stem tissues are mostly associated with pectin and hemicellulose remodeling (Roach et al., 2011; Chabi et al., 2017). Taking into account that the bast fibers contain up to 78% of cellulose (Thygesen et al., 2011), and tertiary cell wall of flax is in fact a composite containing mostly cellulose and pectin and hemicellulose (Gurjanov et al., 2008; Mohnen, 2008; Mikshina et al., 2013), we could hypothesize that pectin and hemicellulose remodeling could represent a biochemical basis for the variation of morphometric and mechanical parameters in different flax varieties.

Subsequent sPLS-DA analysis of the transcriptomic data identified 4 distinct variety clusters and showed that 56 genes corresponded to the first 3 X-variates and explained 73% total variance. Calculations of the average morphometric data for the four clusters revealed statistically significant differences between them, suggesting a possible link between the identified DEGs and morphometric data. Somewhat surprisingly, only 1 out of the 56 candidate genes, *Lus10037377* encoding a xyloglucan endotransglucosylase/hydrolase 32, could be directly related to cell wall organization supporting our hypothesis that other molecular processes may be important in determining key fiber parameters.

The calculation of Pearson correlation coefficients enabled the identification of 5 (out of 56) genes showing a strong correlation between expression levels and morphometric features. One of the five identified genes – *Lus10022736* (*LusWRKY85*) – showed a strong positive correlation with the number of elementary fibers per bundle. There are 102 *LusWRKY* genes predicted in the flax genome, and the *LusWRKY85* (*Lus10022736*) gene, together with its near paralogues *LusWRKY84 (Lus10014177)*, *LusWRKY87 (Lus10025133)* and *LusWRKY88 (Lus10025216)* are included in the same group of transcription factors (group III), which is distinguished on the distribution of conserved motifs in *LusWRKY*-genes (Yuan et al., 2021). Developmental studies have shown that *LusWRKY85* (*Lus10022736*) is expressed mostly at the seedling and maturity stage (Yuan et al., 2021). Since the number of elementary fibers per bundle is largely determined by their intrusive growth (Mokshina et al., 2018), it is tempting to hypothesize that the observed expression profile of this gene at the early stages of development could explain the correlation with this trait. Another gene *LusS40-6* (*Lus1000207*3) encodes a senescence regulator and shows a strong statistically significant negative correlation with both of the measured morphometric features. Members of the *S40* gene group were described in barley and *Arabidopsis* plants as playing a role in regulation of leaf senescens (Krupinska et al., 2002; Fischer-Kilbienski et al., 2010), and it is possible that its low expression may lead to a delay in senescence, thereby allowing for increased accumulation of cellulose-rich cell wall material and enhanced cell surface.

In order to identify additional actors of fiber development process and/or confirm transcripts identified via transcriptomics analyses, comparative proteomics were performed on the two varieties Diane (Spring fiber) and Oliver (Winter oil) that belonged to the most widely separated clusters identified both during transcriptomic and morphometric analyses. After analysis, 32 non-redundant proteins were identified, of which 15 and 17 were more abundant in Diane and Oliver, respectively compared to each other. None of the identified proteins were common with the 56 genes identified in the first 3 X-variates of the transcriptomic data. Such an observation is not unusual since protein abundance is not only determined by transcript abundance but also by other factors, including translation efficiency and protein turnover, as well as by a wide range of post-translational modifications (Gygi et al., 1999; Haider and Pal, 2013). Nevertheless, 5 of the proteins were also present in the 1041 DEGs identified by transcriptomics (*Lus10023569*, *Lus10014003*, *Lus10037377*, *Lus10001508*, and *Lus10030577*).

To evaluate the relative weight of the 32 identified proteins as potential markers for fiber-related parameters in the complete panel of 7 varieties, we used PLS-DA to generate clusters based on the transcript levels of the corresponding genes. This was done since insufficient proteomic data was available for sPLS-DA. Hierarchical clustering identified 3 clusters that more accurately reflected the variety morphotype (spring-/winter-fiber and oil) than similar clustering based on the 1041 DEGs. A similar clusterization was obtained for the same 7 flax varieties based on retrotransposon-derived polymorphism (Galindo-González et al., 2016). This study also revealed that the Ty1-copia retrotransposons have been active since breeding started in flax, and that they decreased the expression level of some genes.

Calculation of Pearson correlation coefficients between the expression levels of the genes corresponding to the 32 identified proteins and morphometric data led to the identification of 4 genes showing significant negative correlations. Two of the genes (*Lus10010846* and *Lus10024383*) corresponded to the same *Arabidopsis thaliana* orthologue (*AT2G37660*), which encodes a NAD(P)-binding Rossmann-fold superfamily protein, also referred to a putative 3-β-hydroxysteroid dehydratase/isomerase. This multifunctional steroid metabolism enzyme is suspected to be involved in antioxidative defense, protein folding, and repair in various subcellular locations such as the apoplast, chloroplast, chloroplast stroma, endoplasmic reticulum and thylakoid. Interestingly, certain *Arabidopsis* sterol biosynthesis mutants show crucial alterations in plant organization at the cellular level in globular stage embryos, including incomplete cell wall formation and aberrant cell wall thickening (Schrick et al., 2004). The authors hypothesize that sterols could be crucial for cellulose synthesis during cell wall construction. Another example of a sterol-related enzyme is GAME25 that plays a key role in the formation of steroidal specialized metabolites (Sonawane et al., 2018). Its activity not only affects the enormous diversity of steroidal glycoalkaloids and steroidal saponins, which are produced in hundreds of plant species but also modulates the molecules’ toxic effects. It is interesting to note that this protein has been previously referred to as a lipid-binding protein in phloem exudates of *Arabidopsis* (Guelette et al., 2012) and that its gene is co-expressed with *Lus10006497* coding a Lipase/lipooxygenase, involved in mediating response to stresses such as pathogen infection (Ascencio-Ibáñez et al., 2008). Another highlighted gene is *Lus10041602* encoding an RNA-binding (RRM/RBD/RNP motifs) family protein such as PLASTID-SPECIFIC RIBOSOMAL PROTEIN 2, a protein unique to plastid ribosomes (Yamaguchi and Subramanian, 2003). Transgenic *Arabidopsis* plants overexpressing PSRP2 showed delayed germination compared with that of wild-type plants under salt, dehydration, or low-temperature stress conditions (Tiller et al., 2012). The PSRP2 protein shows RNA chaperone activity, and taken together these results could suggest that PSRP2 may play a role as a negative regulator in seed germination (Xu et al., 2013). The fourth gene (*Lus10008932*) encodes a cytosolic or nuclear polyketide cyclase/dehydrase and lipid transport superfamily protein, also known as MLP-like protein 328 (Major Latex protein-). A previous study showed that MLP-like protein 328 transcripts were the most abundant in flax stem tissues above the snap point (Guo et al., 2021), which is the transition zone in development process of fiber cells (Gorshkova et al., 2003).

Our attempts to link the Omics results with morphometric data were based upon analyses of living plant material harvested at the budding stage. As such, we considered the morphometric data as *in-planta* factors. However, as explained above, key fiber parameters (fineness, mechanical properties) are also affected by fiber separation and extraction during the post-harvest retting process (Djemiel et al., 2020). Since these actions occur after the plant has been harvested, we decided to refer to the obtained data as *ex-planta*. In order to see whether these two sets of data were related, linear regressions were made between each of the four fiber bundle quality properties (dependent variable) with the two morphometric parameters (independent variables). The results showed that the studied morphometric features were able to explain close to 50% of the Young’s modulus variation and about 30% of the strength variation. This fact suggests that the measurements of the cell surfaces and the number of elementary fibers per bundle before retting could be considered as relevant indicators of the biological background that influence the mechanical properties of the fiber bundle after retting and scutching. It also suggests that it is necessary to consider both *in-planta* and *ex-planta* properties to be able to explain fiber variability especially when considering the Young’s modulus, which is a relevant indicator of the biological quality of a fiber bundle (Haag and Müssig, 2016).

Calculation of Pearson correlation coefficients led to the identification of 3 genes whose expression levels within bast fiber bearing tissues showed a strong correlation with *ex-planta* mechanical parameters of scutched fibers obtained after retting. Similar approach using transcriptomic data and *ex-planta* parameters was recently described for flax (Galinousky et al., 2020) which shown comparable explanatory potential of individual molecular markers for mechanical features of the scutched fibers (up to 54% described by Galinousky et al., 2020; and 48-54% in the current study). In our study we highlighted the *Lus10011125* gene that encodes an enzyme that catalyzes the oxidative decarboxylation of (S)-malate to pyruvate using NADP+ as a cofactor. According to the FIBexDB^9^ data, the expression level of this gene increases in flax roots treated with *Fusarium oxysporum* or aluminum, and also in the reduced fiber flax mutant (the rdf-mutant) (Dmitriev et al., 2016; Dmitriev et al., 2017; Mokshina et al., 2021; Petrova et al., 2022). The expansin-like protein encoding gene (*gi|171462119*) whose expression strongly correlates with the maximum force, may regulate the mechanical properties of flax fibers by affecting the degree of cellulose microfibril alignment and the interactions between cellulose and other cell wall components (Cosgrove, 2000). At last, *Lus10010846*, coding a binding Rossman-fold superfamily protein involved in biosynthesis and maintenance of plant cell walls, was already previously shown to be highly correlated with morphometric features (**Figure 6**), and could be of great interest for both *in-planta* and *ex-planta* fiber features management and prediction.

## 5 Conclusion and future perspectives

Our study indicates that Omics combined with appropriate statistical analyses can be a powerful tool for identifying genes potentially involved in determining *in-planta* and *ex-planta* properties related to flax fiber quality. The results suggest that the observed differences in morphometric properties between the studied varieties have a background in differentially-expressed genes involved in different biochemical pathways. The genetic variability of the studied varieties may be reflected in the functioning of a sub-set of key genes present in the bast fiber bearing tissues, including those involved in lipid metabolism in the phloem and senescence process. The expression of specific lipid transfer proteins was previously correlated with either the elongation or thickening stage of flax bast fiber development (Roach and Deyholos, 2007). Similarly, previous expression data showed that about 11% of over-expressed genes in the bast of the stem were potentially associated with lipid and wax metabolism (Fenart et al., 2010). The association of delayed senescence processes and intensive formation of bast fibers could be related to the later transition of flax fiber varieties to budding and flowering compared to oil seed, as well as to their more prolonged rapid growth stage and higher stem length (Diederichsen and Richards, 2003). There is also increasing evidence that remodeling of cell wall polymers is an important process during the formation and maturation of flax fibers. Molecular actors involved in cell wall remodeling may therefore represent a potential target for improving flax fiber properties.

Genomic breeding approaches commonly include the development of an optimal genomic model which utilizes a set of genetic markers favorable for the desired phenotypic trait (He et al., 2019; Khan et al., 2023; Rahman and Hoque, 2023). In our current study, we show how individual protein and RNA markers have a higher explanatory potential for phenotypic features and potentially could be used as effective predictors of flax fiber quality parameters. In the context of the global climate change trend, these genes we have highlighted could be tested over several years and under different climatic conditions.

Overall, our results suggest that the range of possible molecular processes that could be targeted for genetic improvement of flax fiber quality is far larger than previously imagined above and beyond the different metabolisms associated with the biosynthesis of cell wall polymers. We hypothesize the development of a new generation of breading approaches based on the combined use of Omic data and appropriate bio-informatics tools.

## Data availability statement

The transcriptomic data presented in the study are deposited in the NCBI repository, accession number GSE222066. The proteomics data presented in the study are deposited in the ProteomeXchange repository, accession number PXD039389.

## Author contributions

MC, SH, GN, AD, A-SB, BC, and SG contributed to conception and design of the study. SH secured the funding. MC conducted the experiment. KH and JM conducted the mechanical analyses. MC, JR, and SP conducted the proteomic analysis. MC and AL-D conducted the transcriptomic analyses. EG and DG performed the Omics statistical analyses. MC and SH wrote the first draft of the manuscript. EG, DG, GN, SH, AD and BC wrote sections of the manuscript. All authors contributed to manuscript revision, read, and approved the submitted version.

## Footnotes

1
https://imagej.net/software/fiji/

2
https://phytozome-next.jgi.doe.gov

3
http://cran.r-project.org/

4
https://digitalinsights.qiagen.com/

5
https://www.ebi.ac.uk/pride/archive

6
http://bioinformatics.sdstate.edu/go/

7
https://www.plabipd.de/portal/web/guest/mercator4

8
https://phytozome-next.jgi.doe.gov

9
https://ssl.cres-t.org/fibex/

10
http://bioinformatics.sdstate.edu/go74/

11
https://software.broadinstitute.org/morpheus/

12
https://software.broadinstitute.org/morpheus/
