# Supplementary figures and images for: Identification of new potential molecular actors related to fiber quality in flax through Omics
Source: Front Plant Sci. 2023 Jul 17;14:1204016. doi: 10.3389/fpls.2023.1204016 (PMC10390313; doi:10.3389/fpls.2023.1204016)

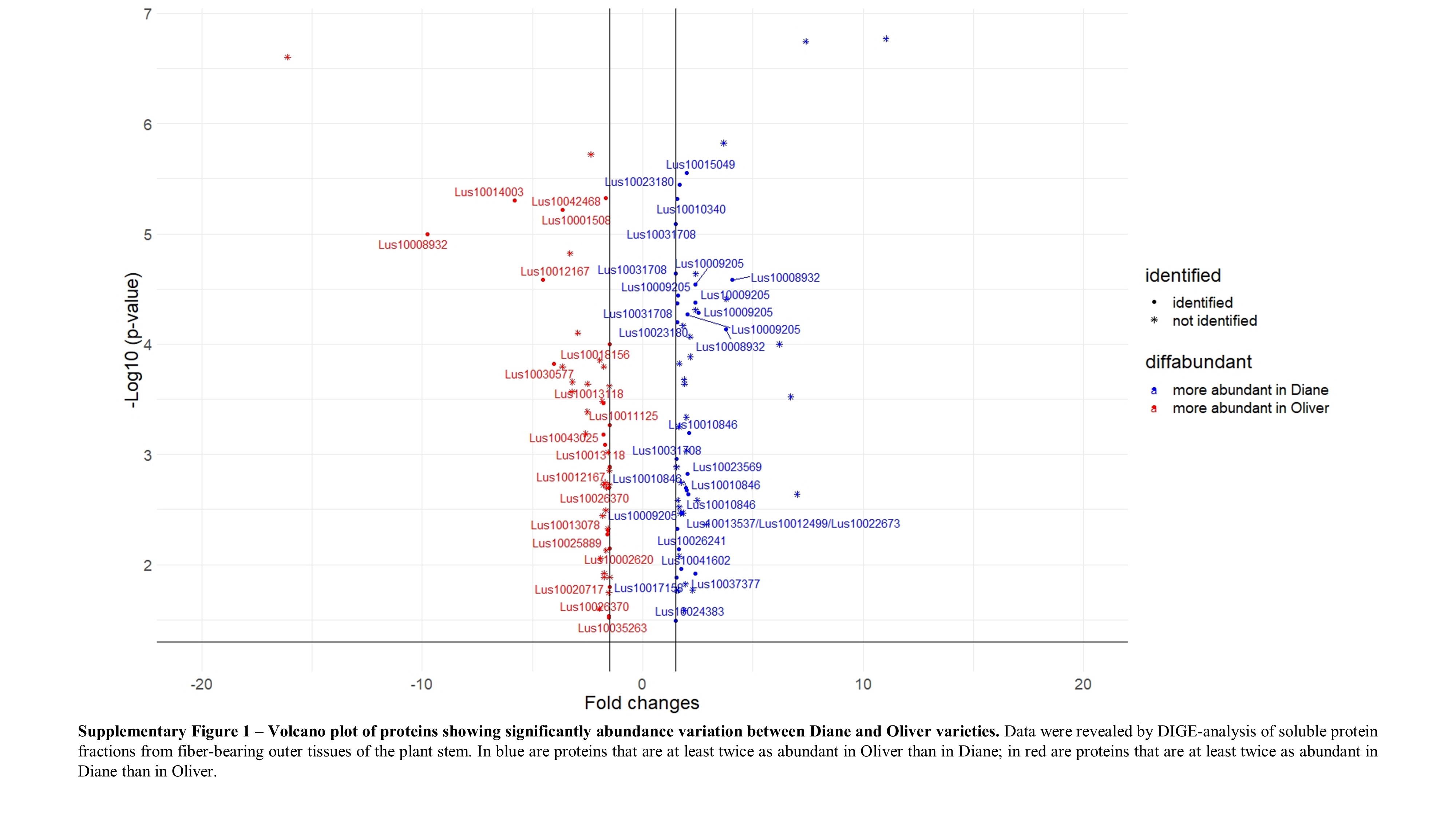

Supplement: Supplementary file 1 [file DataSheet_1.zip › Supplementary materials/Supplementary Figure1.jpg]
